# Supplementary material for: Autoclave treatment of the classical scrapie agent US No. 13-7 and experimental inoculation to susceptible VRQ/ARQ sheep via the oral route results in decreased transmission efficiency
Source: PLoS One. 2020 Dec 3;15(12):e0243009. doi: 10.1371/journal.pone.0243009 (PMC7714121; doi:10.1371/journal.pone.0243009)
Supplement: S1 Fig — (PDF) [file pone.0243009.s001.pdf]

S1. western blot used to make figure 3
Sheep 3807 - classical scrapie pos control
sheep 288 - classical scrapie - autoclaved group - pos
Sheep 242 - classical scrapie - non-autoclaved - pos
Sheep 243 - scrapie negative - autoclaved group - neg

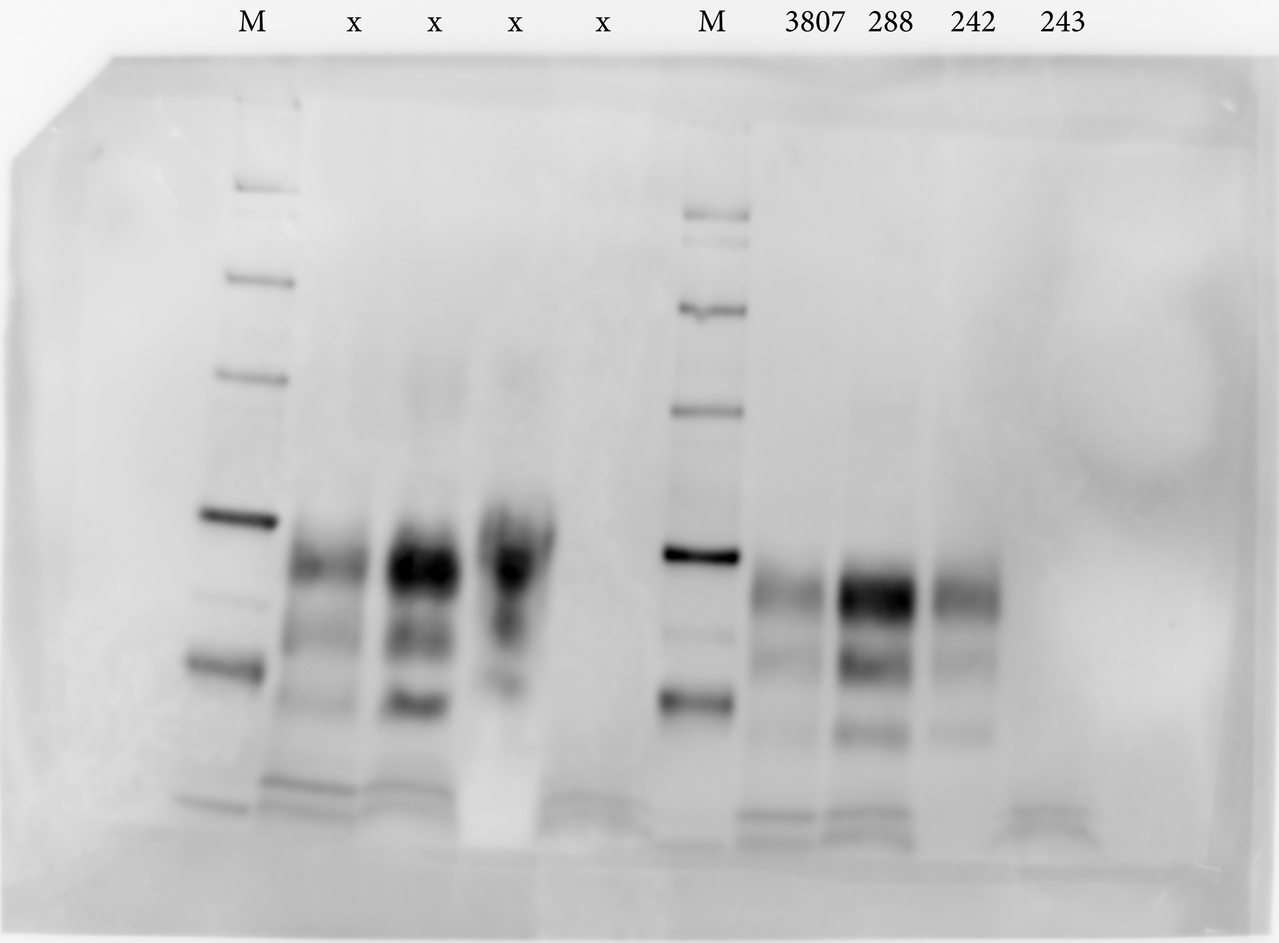

Captured with G:Box (synoptics) Blue LED module - Filter SW06 - 4.5398 second exp
