# Supplementary material for: Autoclave treatment of the classical scrapie agent US No. 13-7 and experimental inoculation to susceptible VRQ/ARQ sheep via the oral route results in decreased transmission efficiency
Source: PLoS One. 2020 Dec 3;15(12):e0243009. doi: 10.1371/journal.pone.0243009 (PMC7714121; doi:10.1371/journal.pone.0243009)
Supplement: Attachment — Submitted filename: Response to Reviewers.docx [file pone.0243009.s002.docx]

Dear Dr. Greenlee,

Thank you for submitting your manuscript to PLOS ONE. I apologize for the prolonged review process; it was difficult to collect reviews in these crazy times. After careful consideration, we feel that it has merit but does not fully meet PLOS ONE’s publication criteria as it currently stands. Therefore, we invite you to submit a revised version of the manuscript that addresses the points raised during the review process.

As listed below, the reviewers expressed some concerns and suggested ways to improve your manuscript. Among the key concerns that I would ask you to consider are insufficient clarity as to 1) why you chose those particular autoclaving conditions for your study, and 2) the strength of your conclusions in the absence of more direct comparisons of your treatment conditions to other conditions, or, your chosen scrapie strain to other strains. One reviewer felt strongly enough about this to recommend rejection. Also there is the issue of the staining in Fig 2A as well as other issues that are worth serious consideration.

Please submit your revised manuscript by Nov 19 2020 11:59PM. If you will need more time than this to complete your revisions, please reply to this message or contact the journal office at [plosone@plos.org](mailto:plosone@plos.org). When you're ready to submit your revision, log on to <https://www.editorialmanager.com/pone/> and select the 'Submissions Needing Revision' folder to locate your manuscript file.

Please include the following items when submitting your revised manuscript:

- A rebuttal letter that responds to each point raised by the academic editor and reviewer(s). You should upload this letter as a separate file labeled 'Response to Reviewers'.
- A marked-up copy of your manuscript that highlights changes made to the original version. You should upload this as a separate file labeled 'Revised Manuscript with Track Changes'.
- An unmarked version of your revised paper without tracked changes. You should upload this as a separate file labeled 'Manuscript'.

Journal Requirements:

When submitting your revision, we need you to address these additional requirements.

1. Please ensure that your manuscript meets PLOS ONE's style requirements, including those for file naming. The PLOS ONE style templates can be found at

<https://journals.plos.org/plosone/s/file?id=wjVg/PLOSOne_formatting_sample_main_body.pdf> and

<https://journals.plos.org/plosone/s/file?id=ba62/PLOSOne_formatting_sample_title_authors_affiliations.pdf>

Response: The files were renamed.

2. To comply with PLOS ONE submissions requirements, in your Methods section, please provide additional information on the animal research and ensure you have included details on (1) methods of sacrifice, (2) methods of anesthesia and/or analgesia, and (3) efforts to alleviate suffering.

 Response: This information was added to the methods (lines 88-89 and 96).

3.. PLOS ONE now requires that authors provide the original uncropped and unadjusted images underlying all blot or gel results reported in a submission’s figures or Supporting Information files. This policy and the journal’s other requirements for blot/gel reporting and figure preparation are described in detail at <https://journals.plos.org/plosone/s/figures#loc-blot-and-gel-reporting-requirements> and <https://journals.plos.org/plosone/s/figures#loc-preparing-figures-from-image-files>. When you submit your revised manuscript, please ensure that your figures adhere fully to these guidelines and provide the original underlying images for all blot or gel data reported in your submission. See the following link for instructions on providing the original image data: <https://journals.plos.org/plosone/s/figures#loc-original-images-for-blots-and-gels>.

Respose: The uncropped image is uploaded and available for review as supporting information.

In your cover letter, please note whether your blot/gel image data are in Supporting Information or posted at a public data repository, provide the repository URL if relevant, and provide specific details as to which raw blot/gel images, if any, are not available. Email us at [plosone@plos.org](mailto:plosone@plos.org) if you have any questions.

4. Please include captions for your Supporting Information files at the end of your manuscript, and update any in-text citations to match accordingly. Please see our Supporting Information guidelines for more information: [http://journals.plos.org/plosone/s/supporting-information](about:blank).

Response: Captions have been added.

Reviewers' comments:

Reviewer's Responses to Questions

**Comments to the Author**

1. Is the manuscript technically sound, and do the data support the conclusions?

The manuscript must describe a technically sound piece of scientific research with data that supports the conclusions. Experiments must have been conducted rigorously, with appropriate controls, replication, and sample sizes. The conclusions must be drawn appropriately based on the data presented.

Reviewer #1: Yes

Reviewer #2: No

2. Has the statistical analysis been performed appropriately and rigorously?

Reviewer #1: Yes

Reviewer #2: Yes

3. Have the authors made all data underlying the findings in their manuscript fully available?

The [PLOS Data policy](https://gcc02.safelinks.protection.outlook.com/?url=http%3A%2F%2Fwww.plosone.org%2Fstatic%2Fpolicies.action%23sharing&data=02%7C01%7C%7Ce15cb2dc4fba422a1ae108d8695c9b6e%7Ced5b36e701ee4ebc867ee03cfa0d4697%7C0%7C0%7C637375193746839893&sdata=6MX%2BH8yW0gNSuZvum1WtX0cOqI7rMSq2jmco3vIDDWk%3D&reserved=0) requires authors to make all data underlying the findings described in their manuscript fully available without restriction, with rare exception (please refer to the Data Availability Statement in the manuscript PDF file). The data should be provided as part of the manuscript or its supporting information, or deposited to a public repository. For example, in addition to summary statistics, the data points behind means, medians and variance measures should be available. If there are restrictions on publicly sharing data—e.g. participant privacy or use of data from a third party—those must be specified.

Reviewer #1: Yes

Reviewer #2: Yes

4. Is the manuscript presented in an intelligible fashion and written in standard English?

PLOS ONE does not copyedit accepted manuscripts, so the language in submitted articles must be clear, correct, and unambiguous. Any typographical or grammatical errors should be corrected at revision, so please note any specific errors here.

Reviewer #1: Yes

Reviewer #2: Yes

5. Review Comments to the Author

Please use the space provided to explain your answers to the questions above. You may also include additional comments for the author, including concerns about dual publication, research ethics, or publication ethics. (Please upload your review as an attachment if it exceeds 20,000 characters)

***Reviewer #1: Dear Authors,

Your manuscript "Autoclave treatment of the classical scrapie agent US No. 13-7 and experimental inoculation to susceptible VRQ/ARQ sheep via the oral route results in decreased transmission efficiency" was clearly written and the data/conclusion was discussed in a fare and meaningful manner. The experimental design and read-out were quite simple.

A few comments***
 ***1. It would be helpful to include the reasoning on why the specific autoclave parameters for this study were selected (place in intro).***

Response: Thank you for the suggestion. The autoclave parameters were selected because they are standard operating settings. This is different than the APHIS recommended parameters (as discussed in the discussion). Per your suggestion, we clarified in the introduction that the autoclave treatment selected was “standard”.

***2. The text describes the failure to detect PrP in RAMALT biopsies collected from sc+ mice in the group infected with autoclaved tissue. This is interesting, especially since many of the sc+ sheep were still negative even at the time of euthanasia. Can you speculate a reason why you think this is occurring? Were the strain properties/tissue tropisms altered by autoclaving? Is there any precedence for a change in tissue distribution like this following autoclaving or inactivation by other means? (line 213).***

Response: Only 3 sheep had negative antemortem rectal biopsies and a positive final scrapie status (237 and 255 and 270). There were several months (6, 3, and 7 respectively) between the last antemortem rectal biopsies and postmortem analyses on these sheep. We suspect that lower titers in the inocula led to longer incubation periods and slower lymphoid distribution. It’s also possible that IHC simply missed positive follicles (false negative). A difference in lymphotropism is not likely given the widespread (in RPLN, tonsils, spleen and GALT) lymphoid distribution in 4/5 positive sheep. Even sheep 270 without splenic and tonsillar PrPSc had PrPSc in the retropharyngeal lymph node and the small intestinal GALT.

***3. Please provide more detail on how the brain/s was autoclaved. How much mass? Containment? Was it whole brain or already homogenized.***

Response: We have added this information to the materials and methods (lines 80-82).

***4. Figure 1 would depict the data more accurately as a staircase survival curve. As shown, the percentage of sheep alive drops immediately below 100% at day 1. Since the incubation periods are long, and the N values somewhat small in one group, using a staircase style would be preferred.***

Response: We made changes to Figure 1 and redesigned the survival curve. This led to us ascertaining that 5 sheep in the autoclave scrapie group were not included in the first curve. They were added and censored appropriately for surviving to the experimental endpoint. This changed the p-values for our analyses; those results were updated in the manuscript as well.

***Reviewer #2: In the current study, the authors assessed the effect of autoclaving at 121°C for 30 minutes on the infectivity of classical scrapie, the US scrapie strain 13-7. Autoclave treatment prolonged the incubation periods on VRQ/ARQ sheep when orally inoculated with the inoculum. It is somewhat hard to understand the goal of the study. If the authors focus on the effect of autoclave treatment on the infectivity, various condition for autoclave should have been set. If the authors focus on the prion strain, different strains should have been compared. From Fig. 2A, there may be a technical issue of PrPSc detection by IHC on the brain tissue. The immune reactivity is too homogenous.***

Response: We appreciate the suggestions for ways to improve this study. We conducted a simple study in order to answer a simple question. That question was would standard autoclaving alone remove infectivity of classical scrapie in the native host? The reviewer mentioned that we discussed “infectivity” and “strain differences” in our paper. These topics became part of the manuscript’s discussion after evaluating the results of our short experiment. In an attempt at writing a thorough discussion, we mentioned that previous research has shown differences in infectivity between strains after decontamination methods. It’s prudent to mention variables that could possible obscure results between different studies.

In the present experiment, we tested whether or not sheep were orally susceptible to a US classical scrapie strain after standard autoclave parameters. Neither sheep, the native host, nor this strain of scrapie have ever been used in such an experiment before. We appreciate the suggestion to make extra comparisons, but incorporating extra sheep into such a study aimed at answering a simple question would add sizeable expense and duration. The use of the native host species has its benefits, but cost and duration often preclude large study designs testing multiple variables.

A new figure 2 was made that demonstrates the immunoreactivity more clearly. The amount of PrPSc staining in the neuropil observed at low magnification seems homogenous, but at higher magnification (plate C) individual granules and coarse particulate are observed. These staining characteristics are typical of sheep with clinical disease and it is not consistent with technical staining issues.

6. PLOS authors have the option to publish the peer review history of their article ([what does this mean?](https://gcc02.safelinks.protection.outlook.com/?url=https%3A%2F%2Fjournals.plos.org%2Fplosone%2Fs%2Feditorial-and-peer-review-process%23loc-peer-review-history&data=02%7C01%7C%7Ce15cb2dc4fba422a1ae108d8695c9b6e%7Ced5b36e701ee4ebc867ee03cfa0d4697%7C0%7C0%7C637375193746849849&sdata=qUCz%2FI4OH6STHWxr5vLBnYXd8v%2Fi8EzP4p4xQ3%2FV98c%3D&reserved=0)). If published, this will include your full peer review and any attached files.


If you choose “no”, your identity will remain anonymous but your review may still be made public.


**Do you want your identity to be public for this peer review?** For information about this choice, including consent withdrawal, please see our [Privacy Policy](https://gcc02.safelinks.protection.outlook.com/?url=https%3A%2F%2Fwww.plos.org%2Fprivacy-policy&data=02%7C01%7C%7Ce15cb2dc4fba422a1ae108d8695c9b6e%7Ced5b36e701ee4ebc867ee03cfa0d4697%7C0%7C0%7C637375193746849849&sdata=oalcbT5miItRhle3AS8ttse16i1Yr7%2FgslE8I7CTj28%3D&reserved=0).

Reviewer #1: No

Reviewer #2: No

[NOTE: If reviewer comments were submitted as an attachment file, they will be attached to this email and accessible via the submission site. Please log into your account, locate the manuscript record, and check for the action link "View Attachments". If this link does not appear, there are no attachment files.]

While revising your submission, please upload your figure files to the Preflight Analysis and Conversion Engine (PACE) digital diagnostic tool, [https://pacev2.apexcovantage.com/](https://gcc02.safelinks.protection.outlook.com/?url=https%3A%2F%2Fpacev2.apexcovantage.com%2F&data=02%7C01%7C%7Ce15cb2dc4fba422a1ae108d8695c9b6e%7Ced5b36e701ee4ebc867ee03cfa0d4697%7C0%7C0%7C637375193746859805&sdata=RRDCyljo3lISQhYgBGEZludqtU%2BPXKgtpxagakp8Cho%3D&reserved=0). PACE helps ensure that figures meet PLOS requirements. To use PACE, you must first register as a user. Registration is free. Then, login and navigate to the UPLOAD tab, where you will find detailed instructions on how to use the tool. If you encounter any issues or have any questions when using PACE, please email PLOS at [figures@plos.org](mailto:figures@plos.org). Please note that Supporting Information files do not need this step.
